# The Rapidly Expanding Family of Human Polyomaviruses: Recent Developments in Understanding Their Life Cycle and Role in Human Pathology

**DOI:** 10.1371/journal.ppat.1003206

**Published:** 2013-03-14

**Authors:** Martyn K. White, Jennifer Gordon, Kamel Khalili

**Affiliations:** Department of Neuroscience, Center for Neurovirology, Temple University School of Medicine, Philadelphia, Pennsylvania, United States of America; University of Alberta, Canada

## Abstract

Since their discovery in 1971, the polyomaviruses JC (JCPyV) and BK (BKPyV), isolated from patients with progressive multifocal leukoencephalopathy and polyomavirus-associated nephropathy, respectively, remained for decades as the only known members of the Polyomaviridae family of viruses of human origin. Over the past five years, the application of new genomic amplification technologies has facilitated the discovery of several novel human polyomaviruses (HPyVs), bringing the present number to 10. These HPyVs share many fundamental features in common such as genome size and organization. Infection by all HPyVs is widespread in the human population, but they show important differences in their tissue tropism and association with disease. Much remains unknown about these new viruses. In this review, we discuss the problems associated with studying HPyVs, such as the lack of culture systems for the new viruses and the gaps in our basic understanding of their biology. We summarize what is known so far about their distribution, life cycle, tissue tropism, their associated pathologies (if any), and future research directions in the field.

## Introduction

Polyomaviruses belong to a family of small, nonenveloped, DNA tumor viruses, which have small, circular, double-stranded DNA genomes encapsidated in icosahedral virions without a lipoprotein envelope [1]. The family was named from the founding virus, polyoma virus, meaning “many tumors,” which was discovered in the mouse [2], followed later by the prototypical primate polyomavirus, simian virus 40 (SV40), from the rhesus monkey [3]. Two human polyomaviruses (HPyVs) were discovered in 1971 and additional viruses in human were not detected until recent technologies such as digital transcriptome subtraction and rolling circle amplification allowed the identification of eight novel HPyVs over the last five years (Table 1). Polyomavirus JC (JCPyV), one of the first two HPyVs to be discovered, was first isolated from brain tissue of a patient with the CNS demyelinating disease, progressive multifocal leukoencephalopathy (PML) by Padgett et al. in 1971 [4]. This neurotropic virus is now established as the proven causative agent of PML. PML occurs mainly in individuals with highly suppressed immune system function, especially in the context of HIV/AIDS and patients receiving with immunomodulatory therapies for diseases such as MS. PML is very rare despite the high frequency of JCPyV infection indicated by seroepidemiological studies (reviewed in [5]). Polyomavirus BK (BKPyV) [6] was discovered around the same time as JCPyV in a patient with polyomavirus-associated nephropathy (PVAN) in a kidney transplant recipient. Similar to JCV, BKV induces disease in immunosuppressed individuals and is seen most frequently in patients receiving highly immunosuppressive drugs where it is a leading cause of allograft failure. Like JCPyV, seroepidemiological studies indicate BKPyV infection is widespread but PVAN is relatively rare [7].

**Table 1:** A summary of the human polyomaviruses listed in order of discovery and showing their common names, other names, sources, GenBank RefSeq numbers, and the references for their discovery.

Human Polyomavirus	Other Names^a^	Source	Refseq	Reference
Polyomavirus BK	BK Virus, BKV *BKPyV*; Polyomavirus hominis 1	Kidney transplant recipient	V01108	Gardner et al. 1971 [6] b
Polyomavirus JC	JC Virus, JCV; *JCPyV* Polyomavirus hominis 2	PML patient	J02226, NC_0016999	Padgett et al. 1971 [4] c
Karolinska Institute Polyomavirus	KIV, *KIPyV*	Respiratory Tract	EF127906	Allander et al. 2007 [16] d
Washington University Polyomavirus	WUV, *WUPyV*	Respiratory Tract	EF444549	Gaynor et al. 2007 [17] e
Merkel Cell Carcinoma-Associated Polyomavirus	MCV, *MCPyV*	Merkel Cell Carcinoma	EU375803	Feng et al, 2008 [9] f
Human Polyomavirus-6	*HPyV6*	Normal skin	NC_014406	Schowalter et al. 2010 [27] g
Human Polyomavirus-7	*HPyV7*	Normal skin	NC_014407	Schowalter et al. 2010 [27] g
Trichodysplasia Spinulosa-Associated Polyomavirus	TSV, TSPyV HPyV8	Trichodysplasia Spinulosa	NC_014361	van der Meijden et al. 2010 [8] h
Human Polyomavirus-9	HPyV9	Kidney transplant recipient	NC_015150	Scuda et al. 2011 [37] i
MW Polyomavirus	MWPyV, HPyV10	Healthy Stool from Malawi WHIM patient	JQ898292, JX262162	Siebrasse et al. 2012 [38] j; Buck et al. 2012 [39] j

a ICTV-designated abbreviation is shown in italic [10].

b Polyomavirus BK (BKPyV) was one of the first two human polyomaviruses to be discovered and was first isolated from the urine of a kidney allograft recipient with advanced renal failure by Gardner and coworkers in 1971 [6]. BKPyV causes BKPyV-associated nephropathy (BKVAN) in kidney transplant recipients who receive highly immunosuppressive drugs and is a leading cause of allograft failure.

c In 1971, polyomavirus JC (JCPyV) was discovered about the same time as BKPyV and was isolated from brain tissue of a patient with progressive multifocal leukoencephalopathy (PML) by Padgett and coworkers [4]. This neurotropic virus is now established as the proven causative agent of PML.

d Karolinska Institute polyomavirus (KIPyV) was identified in 2007 using a large-scale molecular virus screening approach to human diagnostic clinical samples of nasopharyngeal aspirates to search for previously unrecognized viruses by Allander and coworkers [16] at the Karolinska Institute in Stockholm, Sweden, which identified a novel polyomavirus, KI polyomavirus (KIPyV).

e Washington University polyomavirus (WUPyV) was also identified in 2007 using a high throughput DNA sequencing approach to a random library generated from a nasopharyngeal aspirate from a 3-year-old child from Australia diagnosed with Pneumonia, at Washington University, St. Louis, Missouri, another novel polyomavirus, WU [17].

f Merkel cell polyomavirus (MCPyV) [9] was identified in 2008 by Feng et al. in a search to identify unknown agents in a rare type of human malignancy, Merkel cell carcinoma (MCC) using digital transcriptome subtraction, which is a bioinformatics method to detect the presence of novel pathogen transcripts by computational removal of host sequences using high-throughput sequencing and comparison to the available high-quality reference genome data of the host.

g Human polyomavirus-6 and -7 (HPyV6 and HPyV7) were identified in 2010 using rolling circle amplification (RCA) technique to isolate circular DNA viral genomes from human skin swabs and are chronically shed from human skin in the form of assembled virions [27].

h In 2010, van der Meijden et al. [8] discovered a new human polyomavirus associated with trichodysplasia spinulosa in an immunocompromised patient.

i In 2011, Scuda et al. [37] in a kidney transplant patient under immunosuppression identified a novel human polyomavirus (HPyV9) by PCR using degenerate primers closely related to the African green monkey-derived lymphotropic polyomavirus in samples from a kidney transplant patient under immunosuppression using generic PCR.

j In 2012, Siebrasse et al. [38] isolated from a stool sample collected from a healthy child from Malawi (MWV). Later in 2012, Buck et al. [39] used RCA on condyloma specimens from patient with warts, hypogammaglobulinemia, infections, and myelokathexis (WHIM) syndrome and identified a virus, HPyV10, with 95%–99% sequence similarity to MWV.

**Figure 1:**
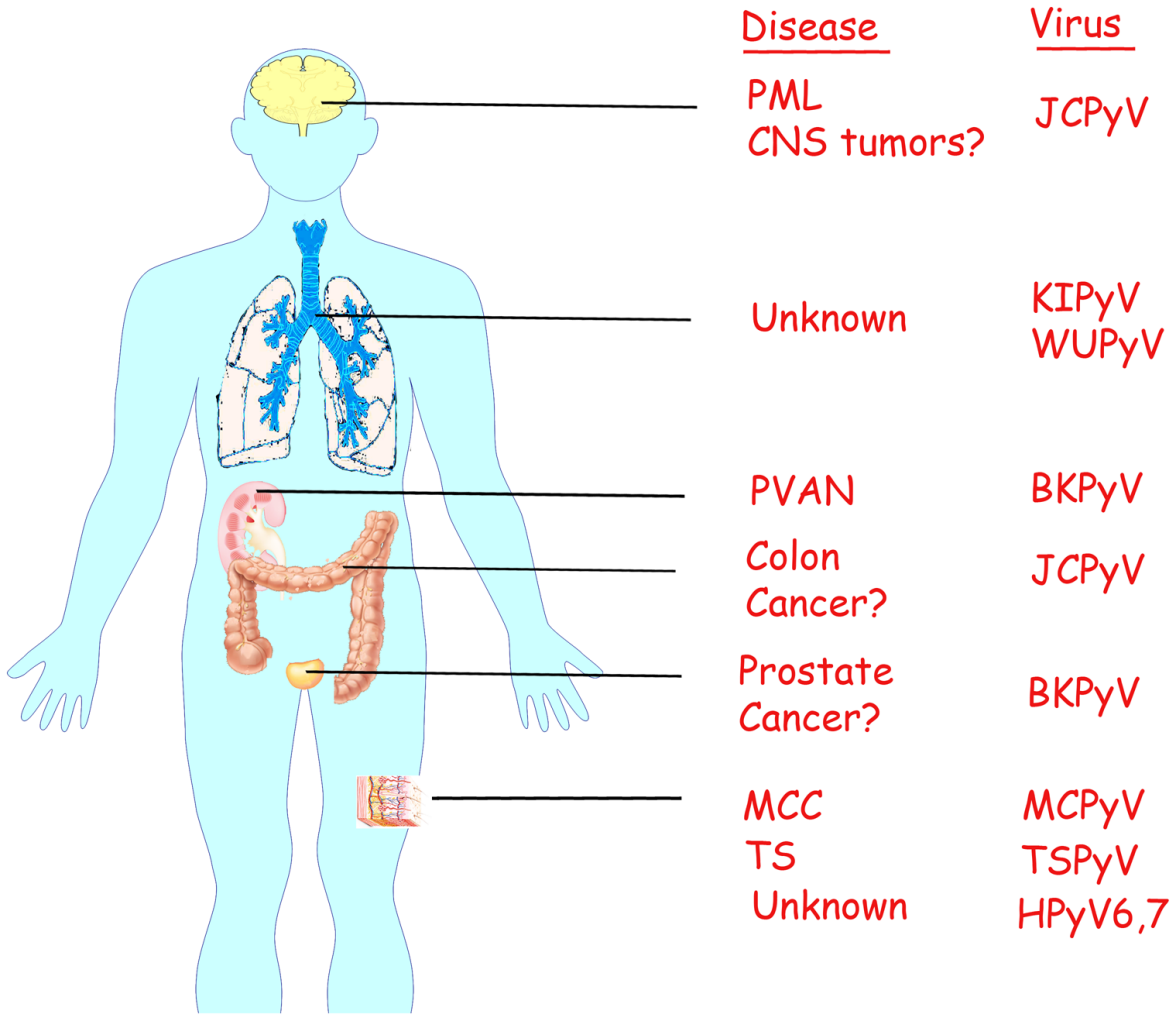
Human polyomaviruses and associated diseases. A schematic representation of the human body showing the organs to which each human polyomavirus has tropism and causes disease.

The human polyomaviruses are important in human pathology for two major reasons. Firstly, while polyomavirus infection is usually asymptomatic, under some circumstances it can be associated with a specific pathology. For example, JCPyV infection and replication in oligodendrocytes of the brain leads to PML, while BKPyV infection and replication in kidney epithelial cells leads to PVAN. Similarly, replication of the polyomavirus, Trichodysplasia Spinulosa virus (TSPyV) [8], in the inner root sheath cells of hair follicles leads to Trichodysplasia Spinulosa (TS), a rare skin dysplasia seen in immunocompromised individuals. Secondly, several polyomaviruses have been shown to encode oncogenic proteins that have transforming ability in cell culture and tumorigenic activity in animals, and thus may have an association with human cancer. As discussed in more detail below, the evidence for such a role in cancer is strongest for Merkel cell polyomavirus (MCPyV) [9], which was first isolated from patients with Merkel cell carcinoma (MCC), a rare and aggressive malignant skin cancer. The involvement of the human polyomaviruses in the pathology of various diseases is illustrated in Figure 1.

## Comparative HPyV Genomics and Phylogeny

**Figure 2:**
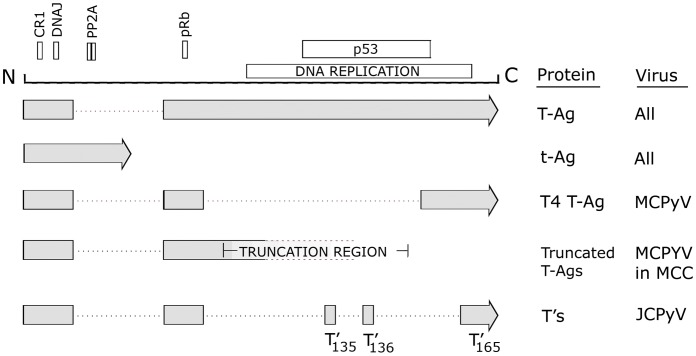
Transforming proteins produced by the early region of human polyomaviruses. A schematic of the early region of human polyomaviruses showing the various protein motifs including conserved region 1 (CR1), the DNAJ domain, the protein phosphatase-2A binding site, the retinoblastoma protein binding site, the region involved in DNA replication, and the p53 binding site. All 10 HPyV produce large T-antigen (T-Ag) and small t-antigen (t-Ag). In addition, MCPyV and JCPyV produce additional splice variants, T4 and the T's, respectively. In Merkel call carcinoma, MCPyV is found integrated in such a way as to cause targeted disruption of T-Ag as indicated by the labeled “truncation region,” which was defined by sequencing the T-Ag genes from multiple MCC (22). Note that truncation at this region preserves the pRb site but not the p53 site or DNA replication region as discussed in the text. The 17kT variants of BKPyV and MCPyV are not shown.

All HPyVs have a similar genome size (4.5–5.4 kbp), organization, and share DNA sequence similarity [1]. The circular genome contains two coding regions, early and late. Transcription of both units is initiated from a common bi-directional regulatory region at the origin of DNA replication (Ori) in opposite directions. The early region encodes the alternatively spliced transforming proteins, large T antigen (T-Ag) and small t antigen (t-Ag), as well as some large T-antigen variants that have so far only been demonstrated for BKPyV, JCPyV, and MCPyV [1] as shown in Figure 2, while the late region encodes the capsid structural proteins VP1, VP2, and VP3. In the case of JCPyV and BKPyV but not the other human polyomaviruses, the late region also encodes a small regulatory protein known as agnoprotein.

The Polyomaviridae family of viruses was until recently comprised of a single genus, Polyomavirus, containing the mammalian and the avian polyomaviruses [10]. Between 2007 and 2012, eight new HPyVs have been discovered (Table 1). In 2010, the Polyomaviridae study group of the International Committee on Taxonomy of Viruses (ICTV) recommended revision of the Polyomaviridae family by the division of the Polyomavirus genus into three genera: Orthopolyomavirus, containing the “classic” mammalian polyomaviruses (e.g., JCPyV, BKPyV, SV40, mouse polyomavirus, etc.); Wukipolyomavirus, containing the novel human polyomaviruses including the Karolinska Institute polyomavirus (KIPyV) and the Washington University polyomavirus (WUPyV); and Avipolyomavirus containing the avian polyomaviruses [11]. This reflects the fact that the orthopolyomaviruses are much more closely related phylogenetically than they are to the wukipolyomaviruses or the avipolyomaviruses.

The DNA sequences of the HPyVs are informative since we can infer that regions which are highly conserved are likely to have shared functionality. For example, the sequences of T-Ag of all HPyV contain predicted p53-binding, pRb-binding, and DnaJ motifs [12]. Similarly, the predicted overall structures of all HPyV VP1 capsid proteins contain similar exposed loops. This is a notable area of active HPyV research, and solved VP1 structures have now been published for JCPyV, MCPyV, WUPyV, and KIPyV, which will expand our knowledge about cellular receptor usage by the HPYV.

Unlike the orthopolyomaviruses JCPyV and BKPyV, the wukipolyomaviruses lack an open reading frame for agnoprotein in the late region. Since agnoprotein has important functions in the life cycle of the orthopolyomaviruses [13], this may indicate an important biological difference between the two genera. It is unusual that agnoprotein is restricted to the orthopolyomaviruses in humans, since it is found in some other mammalian polyomaviruses and indeed in avian polyomaviruses [13] and is thus a relatively evolutionarily old protein. From the study of JCPyV and SV40, it has been found to function in several capacities in facilitating the viral life cycle and modulating host cell processes [13], and these functions must be fulfilled by other mechanisms in the polyomaviruses that lack agnoprotein. These and other findings from genome analysis and their significance have been discussed in detail in two excellent recent reviews [12], [14].

## HPyV Epidemiology and Seroepidemiology

A common feature of HPyV is that the occurrence of viral-associated disease in the population is very rare and yet antibodies to virus can be detected in a large percentage of people, indicating that infection is widespread. For example, most people have antibodies to BKPyV and JCPyV by the second decade of life, but viremia is rarely detected except in patients with PVAN and PML, respectively. Viruria is somewhat less rare but can occur episodically and at low levels in normal people. JCPyV and BKPyV viruria can be increased by immunosuppression, with increased shedding during third trimester pregnancy and in the elderly, for example, though in most cases it remains idiopathic [15]. Like JCPyV and BKPyV, the polyomaviruses KIPyV and WUPyV are also rarely detected and do not seem to be associated with any pathology. They were first amplified in a small fraction of clinical samples of mucus from infants with respiratory disease [16], [17], but their presence here appears to be incidental [18]. Also like JCPyV and BKPyV, their detection frequencies are increased by immunosuppression [19], and seroepidemiological studies show infection to be widespread. In adults over 21, seropositivity rates for KIPyV and WUPyV have been reported to be 55% and 69%, respectively [20], while a study of different age groups reported increases in rates from ages 1 to 79 years, reaching maximum respective rates of 70% and 80% [21]. MCPyV is associated with a rare but aggressive type of human malignancy, Merkel cell carcinoma, where signature events are its clonal integration into the cell genome and T-antigen mutation [9], [22]. Details of the biology of the free virus are currently emerging [23], and this virus also is found to be widespread in seroepidemiological studies [20], [24]. TSPyV causes the rare disease Trichodysplasia spinulosa and has a seroprevalence of 70% [25], [26]. A pilot serological study using ELISA with VP1 VLPs has reported that infection or coinfection with the MCPyV, HPyV6, and HPyV7 polyomaviruses is very common [27]. HPyV9 is cross-reactive with simian lymphotropic polyomavirus and also has a high seroprevalence [28], [29].

## Viral Latency/Persistence, Replication, and Reactivation of Human Polyomaviruses

### Viral Persistence

The observations noted above, that the presence of HPyV is rare while exposure is frequent, have led to the widely accepted paradigm that infection occurs early in life, leading to a primary viremia following which virus is able to enter a state of latency and avoid the attention of the immune system. The site and molecular nature of this viral latency/persistence is poorly understood and likely varies between viruses. In the case of JCPyV, virus is thought to persist in a number of organs including the kidney, bone marrow, and brain (reviewed in [5], [30]). In the kidney, JCPyV is likely undergoing active asymptomatic replication at a low level or episodically in the epithelial cells of the kidney tubules as shown by continuous shedding of the same strains of JCPyV [31]. On the other hand, JCPyV detected in the brain is likely to be in a nonreplicating, nontranscribed state within viral chromatin—that is, silent episomal circular viral DNA in association with histone proteins—since JCPyV DNA can be detected in the absence of viral proteins in normal brain tissue [32]. Unlike JCPyV in the kidney, this may be considered to be true latency—that is, virus production at even a low level has ceased. Similarly, BKPyV may reside as a persistent low-level infection in the kidney in the absence of PVAN since it can also be detected in urine [33]—for example, asymptomatic BK polyomaviruria is well documented in pregnancy [34]. In contrast to JCPyV and BKPyV, KIPyV and WUPyV are not found in urine but are found in nasophayngeal aspirates, bronchoalveolar lavages, and feces of pediatric patients [35], indicating an apparent tissue tropism of these viruses for respiratory epithelia. While KIPyV and WUPyV have been found in samples of mucus from infants indicative of this tropism [16], [17], there are no reports of pathology associated with their presence here, which appears to be incidental [18]. A recent detailed study of pediatric patients was able to ascribe the presence of virus in feces to the swallowing of virus [36]. The other HPyVs also show highly specific tropisms. TSPyV has a tropism for the inner-root sheath cells of hair follicles [8], while MCPyV has a tropism for the neuroectodermal Merkel mechanoreceptor cells of the skin since it is associated with MCC, a tumor that arises from these cells. As discussed below, the finding of virions for MCPyV, HPyV6, and HyPV7 in skin samples represents powerful evidence for the skin tropism of these viruses. At the present point in time, the tropism of HPyV9 [37] and HPyV10 [38], [39] remains unknown.

### Replication and Productive Infection

As described above, polyomaviruses may persist in host cells in the absence of viral replication—that is, a state of latency where virus production at even a low level has ceased, for example JCPyV in the brain. Alternatively, it may persist in a state of active but asymptomatic replication occurring at a low level or episodically, for example JCPyV in the kidney. We define reactivation as the emergence of virus from a persistent or latent state to one of active replication, which causes a pathological condition. In the case of JCPyV, pathology (PML) occurs in the brain and so reactivation likely involves the re-initiation of a productive infection cycle from the latent state. Two features distinguish replicating virus (whether asymptomatic or pathological)—the visualization of virions by electron microscopy (EM) and detection of the expression of the late capsid proteins such as VP1. For JCPyV, the first EM studies to show viral particles in the nuclei of oligodendrocytes of PML lesions were reported in the 1960s [40]–[42]. Expression of JCPyV late capsid protein, VP1, is clearly observable by immunohistochemical labeling in nuclei of oligodendrocytes and astrocytes in PML lesions [43]. The presence of virions is also observed by EM in these cells [44]. For BKPyV, the original isolation of the virus originated from the observation of papovavirus-like particles by EM in the urine cytology of a Sudanese kidney transplant patient (initials, B.K.) with chronic pyelonephritis [6]. Interesting, first-hand accounts of the discovery of BKPyV and JCPyV virions have been published [33], [45]. BKPyV inclusion bodies and viral protein expression can be detected in the kidney epithelium of PVAN patients [46].

KIPyV and WUPyV were identified with a high throughput DNA sequencing approach to a random library generated from nasopharyngeal aspirates of children with respiratory ailments [16], [17]. It was not addressed in the original papers whether the virus replicates in the respiratory system. Since then, these viruses have been detected in other body compartments including blood [47], and their true site of replication remains unknown. For MCPyV, virus associated with MCC is integrated into the host genome, an event not associated with the polyomavirus replicative cycle but has a role in cell transformation as discussed below. MCPyV is difficult to cultivate in tissue culture, with only primary low-level virion production being achieved by transfection/infection and no secondary transmission [23]. Despite early reports of virions associated with MCC in EM, the available evidence suggests that MCPyV does not replicate in MCC [23]. Schowalter et al. [27] provided evidence for the shedding of assembled virions of MCPyV, HPyV6, and HyPV7 using ultracentrifugation of nuclease-treated skin swab specimens under nondenaturing conditions, thus providing evidence for the skin tropism of these viruses. In the case of TSPyV, virus is thought to actively replicate in the inner-root sheath cells in TS, and intracellular viral particles have been detected by EM [48]. No studies have been reported for the other HPyVs.

### Reactivation

The term *reactivation* came to be used during the many years when JCPyV and BKPyV were the only known HPyVs and both viruses were considered to be “slow viruses” due to their slow growth in culture and the long time period between initial infection and subsequent disease development [49]. Polyomaviruses may persist in two distinct nonpathological states—that is, latency or low-level replication, as described above, Therefore, as used here, the term *reactivation* refers to the pathological processes whereby persistent virus emerges to cause disease associated with active viral replication, PML for JCPyV and PVAN for BKPyV. Except for TSPyV, which causes TS, the new HPyVs are not known to cause any disease associated with active replication, and so it is not clear if the term *reactivation* is meaningful or useful, although it is certainly the case that immunosuppression can increase the frequency of detection of these HPyVs as discussed above. Like PML and PVAN, TS appears to result from viral reactivation, which usually occurs in the context of immunosuppression [26], [48]. This includes kidney transplantation [48], [50], chemotherapy for acute lymphocytic leukaemia [48], [51], and chronic lymphocytic leukaemia [52].

### Role of Cell-Mediated Immunity

An important part of the ability of immunosuppression to allow the reactivation of HPyV is thought to be a defect in the ability to detect and eliminate replicating virus, most notably by cell-mediated immunity. Koralnik et al. [53] explored the JCPyV-specific cellular immune responses in patients with PML and control subjects. CD8+ cytotoxic T-cells (CTLs) against JCPyV T-Ag or VP1 were detected in PBMC of HIV-infected PML survivors of PML and an HIV-uninfected patient recently diagnosed with PML. However, a JCPyV-specific CTL response could not be detected in PBMC of HIV-infected PML patients with progressive neurologic disease and an eventual fatal outcome suggesting that JCPyV-specific cellular immunity plays a role in containing PML. In a prospective study, an association was shown between JC virus-specific CTL and early control of PML [54]. The Swiss HIV Cohort Study examined cryopreserved PBMCs and plasma at diagnosis from PML cases (*n* = 29) and three matched controls per case (*n* = 87). It found that PML nonsurvivors had selectively impaired JCPyV-specific T-cell responses compared to CD4+ T-cell-matched controls and failed to mount JCPyV-specific antibody responses [55]. These and other studies highlight the importance of CTL in containing JCPyV replication and hence a mechanism for the reactivation of virus by immunosuppression. Also of note, certain new immunomodulatory therapies are associated with PML, including the monoclonal antibody Natalizumab, which is used in the therapy of Multiple Sclerosis, acts by preventing the extravasation of T-cells, and is associated with PML, suggesting the importance of immunosurveillance in the CNS [56]–[60].

Similarly, CTL responses to BKPyV T-antigen have been examined in patients with PVAN and PML. A CTL response to epitopes shared between JCPyV and BKPyV T-antigen was detected in 77% of healthy individuals as well as in 30% of patients with PVAN and 44% of patients with PML and/or HIV infection [61], [62]. However, CTL responses to BKPyV T-antigen-specific epitopes were lower and only seen in healthy individuals. It should be noted that immune responses to the VP1 capsid proteins of both JCPyV and BKPyV appear immunodominant in normal healthy individuals as well as patients with disease and that a high degree of epitope cross-recognition is seen in the CTL response to VP1 for the two viruses [61], [63]. Thus, the cellular immune response to the VP1 capsid protein is likely to play an important role in controlling polyomavirus reactivation in the normal population as well as in individuals with PML and PVAN.

Although it does not cause a disease associated with viral replication, there have been several studies of immune responses to MCPyV because of its importance in human cancer (see next section). Iyer et al. [64] studied the T-cell response to MCPyV in MCC tumor tissues and blood from MCC patients and controls. Cultured tumor-infiltrating lymphocytes from two out of six MCPyV T-Ag-positive tumors showed a T-cell response to MCPyV T-Ag as assessed by IFN-γ secretion. None of four T-Ag-negative tumors showed a T-cell response to MCPyV T-Ag. Furthermore, a T-cell response to MCPyV T-Ag was also detected in blood from 14 of 27 MCC patients and five of 13 control subjects [64]. Using MCPyV VP1 virus-like particles, T-cell responsiveness to VP1, again measured by IFN-γ secretion, has also been detected in PBMCs from the blood of healthy adults [65], [66] and to the VP1 of TSPyV [66].

As well as a deficit in the ability of the immune response, other factors are likely important in HPyV activation. For PML, we have proposed and provided evidence for a model where an early event in the reactivation of JCPyV involves the transcriptional activation of latent JCPyV DNA in the glial cells of the brain by transcription factors such as NF-κB, NFAT4, and C/EBPβ [67]–[69] that are downstream of proinflammatory cytokines such as TNF-α [70]. Similar mechanisms may also operate for BKPyV [71]. Another factor in JCPyV reactivation may be the HIV-1 transactivator protein Tat. PML occurs most often in the context of HIV-1/AIDS, and Tat has been shown to be a powerful activator of JCPyV transcription [72]. Finally, the observation that PML can occur in patients receiving the therapeutic monoclonal antibody rituximab, which targets B-cells, has led to the speculation that the entry of JCPyV-infected B-cells into the CNS may be important in the reactivation of JCPyV [60].

## HPyV Integration and Pathogenesis: The New Paradigm of MCPyV/MCC

The oncogenicity of the transforming proteins of polyomaviruses, most notably large T-antigen, in cell culture and animal models has been known for decades, and a key concept in this regard has been that of permissivity. When virus enters a cell that is able to support viral DNA replication and late capsid protein expression (i.e., a permissive cell), the result is productive infection. However, when virus enters a cell that is not able to support efficient viral DNA replication and late capsid protein expression but allows expression of the early transforming genes (i.e., a nonpermissive or semipermissive cell), the result is cellular transformation. Nonpermissive situations may arise because the virus is in a cell of a different species (HPyVs only replicate in human cells) or of a different tissue type than the one for which the virus possesses tropism or because of a mutation in T-Ag that disrupts its ability to support DNA replication [9], [22]. The classic example is the transformation of human diploid fibroblasts by SV40 that was first reported in the early 1960s [73], [74]. Despite the cell culture studies, there has been no definitive evidence linking them to the etiology of human cancer until quite recently. For many years, the association of JCPyV with cancer has been reported by many independent laboratories including our own (reviewed in [43], [75], [76]). However, a role for JCPyV in human cancer etiology continues to be debated with no clear consensus being generally agreed upon (for a discussion of the issues involved, see [77] and [78]). Recently, based on “inadequate evidence” in humans and “sufficient evidence” in experimental animals, the WHO International Agency for Research on Cancer Working Group classified BKPyV and JCPyV as “possibly carcinogenic to humans” (Group 2B) [79].

In 2008, the Merkel cell polyomavirus HPyV was discovered clonally integrated into the cell genome in ∼80% of Merkel cell carcinoma (MCC) [9]. MCC is rare but one of the most aggressive forms of skin cancer and is a neuroectodermal tumor that arises from mechanoreceptor Merkel cells [80]. Immunosuppression is a predisposing factor in the development of MCC [81]. Feng et al. [9] studied MCC samples by digital transcriptome subtraction, a bioinformatics method to detect the presence of novel pathogen transcripts by computational removal of host sequences using high-throughput sequencing and comparison to the available high-quality reference genome data of the host. In this way, a fusion transcript was identified between a previously unidentified polyomaviral T-antigen and a human receptor tyrosine phosphatase. Further studies led to the identification of a 5.4 kb novel virus, which was named Merkel cell polyomavirus or MCPyV [9]. Furthermore, MCPyV DNA was clonally integrated into the cell genome, indicating that integration preceded expansion of the tumor, and a metastatic tumor was shown to have the same pattern of integration as the primary tumor [9]. Analysis of other MCC patients has confirmed clonal integration of MCPyV, providing pathological and molecular evidence for a causative role of MCPyV in oncogenesis [82]. Furthermore, knockdown of MCPyV T-Ag expression in MCPyV-positive MCC cell lines using shRNA-expressing vectors induced growth arrest and/or cell death while MCPyV-negative cell lines were unaffected [83]. Since polyomaviruses have no mechanism to excise themselves from the genome, integrated virus can have no part in the virus's replicative life cycle. Interestingly, all integrated MCPyV genomes have mutations that inactivate the helicase activity of T-Ag, and this may prevent autoactivation of integrated virus replication, which could lead to unlicensed initiation of DNA synthesis and DNA damage detrimental to cell survival [22], [84]. As with the other HPyVs, MCPyV also illustrates the theme that the occurrence of infection is widespread in that MCPyV DNA and antibodies can be frequently detected in healthy individuals and tissues, but the disease MCC is rare. Possibly, this reflects the rarity of events that result in the integration of MCPyV DNA in the genome and also result in the targeted disruption of the T-antigen gene in such a way that its transformative properties are retained but its ability to replicate DNA abrogated, with both steps being necessary for the pathogenesis of MCC as well as immunosuppression.

Thus, there is strong evidence that MCPyV is the etiological agent responsible for most cases of MCPyV. Based on the evidence accumulated to date, the WHO International Agency for Research on Cancer recently classified MCPyV as “probably carcinogenic to humans” (Group 2A) [79].

## In Vitro and in Vivo Laboratory Studies

### Cell Culture Models

Chief among the requirements to study the molecular biology of the new HPyV will be the development of model cell culture systems allowing the propagation of virus in tissue culture. In the case of JCPyV, we have developed a primary human fetal astrocyte cell culture system to study the biology of JCPyV [85] and more recently in oligodendrocyte precursor cells [86]. For BKPyV, virus will grow in monkey kidney cell lines such as Vero and CV-1 as well as primary renal proximal tubule epithelial cells [87], [88]. It has also been reported that BKV can productively infect salivary gland cell lines [89] and human endothelial cell lines [90]. Except for JCPyV and BKPyV, there are no culture systems for the growth of the HPyV, and their development should be a priority so as to enable future research on understanding their basic biology. In the case of MCPyV, there have been no successful attempts to cultivate virus in tissue culture [23]. Only primary low-level virion production has been achieved by transfection/infection of 293 cells but with no secondary transmission [91].

### Mechanisms of Cellular Transformation

The early transforming proteins of polyomaviruses (T-Ag and t-Ag) evolved because of the need to activate signaling pathways that drive the cell cycle into S-phase where the components of the host DNA synthesis machinery (e.g., DNA polymerases) are expressed in order to facilitate viral DNA replication. In the abnormal context of a virus entering a nonpermissive cell or of becoming integrated, the activation of these pathways drives the abnormal cell proliferation associated with cellular transformation and tumorigenesis. The mechanisms of transformation by polyomaviruses have been studied for many years and provided a model system for basic research. Indeed the interaction between SV40 T-Ag and the cellular p53 protein was one of the first oncogenic protein–protein interactions to be described [92]. Many other such interactions have been described between the transforming proteins of the “classic” mammalian polyomaviruses, SV40 and JCPyV, and T-Ag has been reported to interact with other cellular proteins including pRb, IRS-1, β-catenin, and others and small t-antigen (t-Ag) with protein phosphatase 2A (PP2A) as we have reviewed previously [75]. The discovery of the role of MCPyV in MCC has led to new studies on the mechanisms of transformation by this virus. It appears that similar mechanisms may be involved; for example, an intact pRb-binding site in MCPyV large T-Ag is required for promoting growth of MCC [93]. However, an intact p53-binding site is not required, suggesting that transformation by MCPyV is p53-independent [22]. MCPyV also expresses a T-Ag splice variant of unknown function, 57kT antigen, which is identical to T-Ag but lacking an origin-binding domain [22]. However, novel mechanisms for MCPyV, which are not seen for SV40 or JCPyV, have also been discovered: T-Ag binding to Vam6p, which disrupts lyosomal clustering [94], and t-Ag binding to and preserving eukaryotic translation initiation factor 4E-binding protein 1 (4E-BP1) hyperphosphorylation, which results in dysregulated cap-dependent translation [95]. Exploring the mechanisms of HPyV transformation remains an active area of research.

### Animal Models

Polyomaviruses in general have a very narrow species host range [1], and HPyVs will only replicate in human cells, thus precluding the possibility of animal models for HPyV replicative diseases. Animal models for tumorigenesis have existed for some time. The polyomaviruses JCPyV, BKPyV, and SV40 form tumors when they are injected into animals or when the early region is introduced into transgenic mice, and we have reviewed these studies previously [75]. Recently, Guastafierro et al. [96] described an early passage MCPyV-positive MCC cell line that can grow when injected subcutaneously into NOD scid gamma mice and forms discrete macroscopic tumors resembling MCC and may provide a useful in vitro model to characterize MCPyV-positive MCC.

## Conclusions and Future Directions

The last five years have seen a rapid expansion of the family of HPyV from 2 to 10 and was facilitated by the advent of novel molecular biological techniques such as rolling circle amplification and digital transcriptome subtraction to detect rare organisms present at low levels. No doubt, more polyomaviruses will continue to be discovered in humans and in the rest of the animal kingdom. The variety and number of polyomaviruses reflects their success in propagating themselves at low levels without causing disease while evading the immune system. This is reflected in the rarity of polyomavirus-associated diseases and their occurrence only in the circumstance of severe immunosuppression. Similarly, the integration of polyomavirus genome into the host cell genome and tumorigenesis is also a very rare event and represents a dead-end in the life cycle of the virus. Like the concept of permissivity, the notion that integration is involved in the process of tumorigenesis by polyomaviruses originated in early cell culture studies with SV40, which showed that viral DNA was integrated into host cell genomic DNA and that the site of integration was apparently random with respect to both the host and the viral DNA [97]–[100]. For MCPyV, the necessity of integration in tumorigenesis is well-documented [9], [22] and was described above in the section on MCC. However, the nature of JCPyV and BKPyV (integrated or episomal) is not well studied in human tumors where association has been reported. Like SV40, integration of JCPyV has been reported in cell culture [101], and this is also the case for tumors arising from injection of virus into the brains of hamsters [102] and owl monkeys [103], although free infectious virus has also been reported in tumors from owl monkeys [104]. Similarly, BKPyV can integrate in cell culture models of transformation [105], [106], although free virus has also been reported in transformed cells [107], [108] and episomal BKPyV has been reported in a human insulinoma [109]. An interesting situation is found for TSPyV in that it causes TS, which is well established to be a dysplasia but not a neoplasia, and free virus is actively replicating since there have been many reports of assembling arrays of virions in EM of TS (see [8] and references therein). Further work will be required to fully elucidate a possible role of integration for JCPyV and BKPyV when they are associated with human tumors.

Thus, the picture emerges that polyomaviruses are usually benign members of the extensive flora of viruses that are associated with the body and that pathology is an accidental rare event that is of no use to the virus. Evolutionarily, polyomaviruses are an ancient family of viruses whose origin goes back to at least the divergence of mammals and birds and probably beyond and the rarity of their involvement in pathology reflects their successful adaption to their hosts.

While many of the new HPyVs have only been very recently discovered and only a handful of studies have been published so far, the field of study of primate polyomaviruses is a very old one and began with the discovery of SV40 over 50 years ago [3]. Indeed SV40 was the workhorse for the earliest work on the molecular biology of important eukaryotic processes including DNA replication, transcription, and cellular transformation, and the complete genome sequence of SV40 was published in 1978 [110], [111]. This work will provide a strong conceptual framework and armamentarium of laboratory techniques for the elucidation of the biology of the new HPyVs. Also of importance are the many studies that have been done on the prototypical HPyVs, JCPyV, and BKPyV since their discovery in 1971 [4], [6]. These have allowed the clarification of the molecular bases for many important aspects of the biology of the viruses including tropism, latency/reactivation, and regulation of replication and transcription, although much more work is needed to better develop a fuller understanding [112], [113].

Clearly, the development of cell culture systems for the newer HPyVs is important to advance research on their biology. If this is difficult or impossible, alternative approaches will have to be adopted. It might be possible to develop an animal model by engrafting human tissue to make “humanized” mice. For example, Matoba et al. [114] studied JCV infection of human cells inoculated into nude mice brains as a model for PML. Alternatively, it may be possible to create cell lines that provide viral proteins in Trans—for example, cell lines that express viral T-Ag may be useful to study DNA replication while cell lines that express viral capsid proteins could be transfected with viral genomic DNA and this might be useful for studying packaging and viral egress from the cell.

The role of the new HPyVs in human cancer (if any) is an important area of future research by screening many different types of cancer using PCR and immunohistochemistry. Except for MCPyV in MCC, none of the new HPyVs has yet to be implicated in human cancer. MCPyV has become well established as a major etiological agent for MCC through a precise application of molecular techniques such as the use of Southern blot to establish clonal integration, digital transcriptome subtraction to verify the expression of T-antigen, and RNA interference to show the essential nature of T-antigen expression. If similar mechanisms of transformation hold for JCPyV and BKPyV, such approaches could be applied to JCPyV and BKPyV to shed light on the long-standing question of their degree of involvement in human cancers. Furthermore, the advances in knowledge about MCPyV have led to new work aimed at the development of diagnostic tests and studies on precise molecular-targeted therapy [23]. In principal, these approaches can be applied to any polyomavirus-associated disease. For example, much like vaccinations against papillomavirus have been used for the prevention of cervical carcinoma, a similar approach against HPyV T-antigen may be possible. Much more needs to be done to further our understanding of HPyVs, and it is indeed an exciting time to work in the field.
